# Elevated intracranial pressure requiring decompressive craniectomy in a child with progressive primary angiitis of the central nervous system: a case report

**DOI:** 10.1186/s13256-021-03005-y

**Published:** 2021-08-06

**Authors:** Lama S. Al-Mansour, Abdulrahman A. AlRasheed, Khaled R. AlEnezi, Hamza M. AlAli

**Affiliations:** 1 Department of Pediatrics, Ministry of National Guards-Health Affairs, Riyadh, Saudi Arabia; 2 Department of Medical Imaging, Ministry of National Guards-Health Affairs, Riyadh, Saudi Arabia; 3 King Abdullah International Medical Research Center, Riyadh, Saudi Arabia; 4 College of Medicine, King Saud bin Abdulaziz University for Health Sciences, Riyadh, Saudi Arabia

**Keywords:** Intracranial pressure, Craniectomy, Primary angiitis, Central nervous system, Case report

## Abstract

**Background:**

Elevated intracranial pressure is a potentially catastrophic complication of neurologic injury in children. Successful management of elevated intracranial pressure requires prompt recognition and therapy directed at both reducing intracranial pressure and reversing its underlying cause. A rare condition that causes elevated intracranial pressure is childhood primary angiitis of the central nervous system, which is a rare inflammatory central nervous system disease that poses diagnostic and therapeutic challenges. To our knowledge, this is the first reported case of angiography-positive progressive childhood primary angiitis of the central nervous system requiring decompressive hemicraniectomy for refractory elevated intracranial pressure in children.

**Case presentation:**

We report the case of a 5-year-old Saudi girl who presented to the pediatric emergency department with fever and new-onset status epilepticus. She had elevated inflammatory markers with radiological and histopathological evidence of angiography-positive progressive childhood primary angiitis of the central nervous system, complicated by elevated intracranial pressure. Despite medical management for both childhood primary angiitis of the central nervous system and elevated intracranial pressure, her neurological status continued to deteriorate and the elevated intracranial pressure became refractory. She developed right uncal, right subfalcine, and tonsillar herniation requiring decompressive hemicraniectomy with a favorable neurological outcome.

**Conclusion:**

Decompressive craniectomy might be considered in cases of angiography-positive progressive childhood primary angiitis of the central nervous system with elevated intracranial pressure refractory to medication. A multidisciplinary approach for the decision of decompressive craniectomy is advised to ensure patient safety and avoid possible morbidities and mortality.

**Supplementary Information:**

The online version contains supplementary material available at https://doi.org/10.1186/s13256-021-03005-y.

## Introduction

Inflammatory central nervous system (CNS) diseases are a rare spectrum of diseases. One such disease is childhood primary angiitis of the CNS (cPACNS), which is described principally in the rheumatological literature. Primary blood vessel inflammation of the brain and/or spinal cord is the hallmark of cPACNS [1–3]. Different phenotypes have been described, including angiography-positive nonprogressive (APNP) and angiography-positive progressive (APP) disease, which affect large/medium-sized vessels, and angiography-negative (AN) disease, which affects small cerebral vessels. Each phenotype has distinct treatment regimens and prognosis [2, 4]. Certain CNS complications of cPACNS include diffused or focal neurological deficits, stroke, impaired vision, and seizures [5]. Elevated intracranial pressure (ICP) is an established phenomenon in children that can lead to overwhelming consequences. Elevated ICP course and management, including decompressive craniectomy (DC), have been well described after failure of medical therapy to control the disease in the setting of traumatic brain injury (TBI) [6]. Elevated ICP in the setting of inflammatory brain diseases has not been reported in children, and only scarcely reported in adults [7]. In cPACNS, there is evidence of vascular inflammation, with elevated proinflammatory cytokines, notably interleukin-1 (IL-1) and tumor necrosis factor (TNF). Both pathological and immunological causes may contribute to elevated ICP [7–9].

We report a rare pediatric case of APP-cPACNS with refractory elevated ICP that required measures for elevated ICP management, including decompressive hemicraniectomy with a favorable neurological outcome.

## Case presentation

A previously medically and surgically healthy 5-year-old Saudi girl presented with neck swelling and fever. She lived with her parents and siblings with good socioeconomic status. Parents are nonconsanguineous. Her initial vital signs during first presentation were a temperature 36.6 °C, blood pressure 85/53 mmHg, heart rate 101 beats/minute, and respiratory rate 22 breaths/minute on room air. She was admitted and diagnosed with right medial clavicular sterile osteomyelitis by magnetic resonance imaging (MRI) and bone scan. She was treated for 2 weeks with intravenous ceftriaxone and vancomycin (see Additional file 1: list of laboratory results and medications). Her symptoms resolved a few days after management initiation, and she was discharged in normal physical and neurological condition with oral sulfamethoxazole–trimethoprim and nonsteroidal antiinflammatory drugs (NSAIDs). Two months after her initial presentation, she was brought again to the pediatric emergency department (PED) febrile, with generalized tonic clonic status epilepticus (SE), which was aborted with lorazepam and phenytoin. She had no prior history of trauma, speech disturbance, limb weakness, behavioral changes, confusion, or febrile convulsions.

In the PED, she was tachypneic, tachycardic, and ill appearing. Her fever was 39 °C, blood pressure 131/94 mmHg, heart rate 142 beats/minute, respiratory rate 36 breaths/minute, and saturation 95% on non-rebreather face mask. Upon physical examination, she exhibited poor neurological responses, with Glasgow Coma Scale (GCS) of 9/15, bilaterally reactive and symmetric pupils, strong gag and cough reflexes, and no focality, or neurocutaneous stigmata. Other systems examination including heart, lung, abdomen, skin, and musculoskeletal system was unremarkable. After SE treatment, she developed hypoventilation requiring intubation and mechanical ventilation.

Initial investigations showed unrevealing extensive infectious work-up including serological and cerebral spinal fluid (CSF) tuberculosis and brucellosis, and noninfectious causes including metabolic, hematological, immunodeficiency, and genetic diseases. Genetic investigations included both patient and her parents, and was nonrevealing, comprising primary immune deficiency (PID) panel and whole-exome and whole-genome sequencing. Immunological investigations comprised immunoglobin levels, oxidative burst assay, and lymphocyte markers. Initial cerebrospinal fluid (CSF) and preliminary inflammatory analyses are summarized in Table 1. Of note, von Willebrand factor (vWF) levels were elevated, and declined with disease improvement. She was started on empirical intravenous broad-spectrum antimicrobial therapy including meropenem, vancomycin, and acyclovir (see Additional file 1: List of laboratory results and medications).

**Table 1:** Laboratory investigations timeline

Examination name	First admission	Upon presentation	One month after presentation	Six months follow-up
CSF protein (g/L)		0.43	0.28	
CSF lactic acid (mmol/L)		1.65	1.56	
CSF glucose (mmol/L)		4.1	4.2	
Appear CSF		Clear	Clear	
Color CSF		Colorless	Colorless	
RBC CSF (10^9^/L)		1	< 1	
WBC CSF (10^9^/L)		1	< 1	
CSF lymphocytes (%)		Rare	None seen	
CSF monocytes (%)			None seen	
CSF segmental (%)		Rare	None seen	
VW factor (%)		> 199.5	> 199.5	125.9
CRP (mg/L)	59	106	14	5
ESR (mm/hour)	120	47	19	30

*CSF* cerebrospinal fluid, *RBC* red blood cell, *WBC* white blood cell,* VW* von willebrand, *CRP* C-reactive protein, *ESR* erythrocyte sedimentation rate

Initial brain computed tomography (CT) revealed no acute brain insult, and initial electroencephalogram (EEG) showed diffused nonspecific slowing with no epileptiform discharges. She was then shifted to the pediatric intensive care unit (PICU).

On hospital day (HD) 1, the patient’s course was complicated with increased right focal seizures and signs of elevated ICP (fixed dilated pupils, bradycardia, and hypertension). She was managed with hyperventilation, 3% intravenous sodium chloride (NaCl) 5 mL/kg, and intravenous mannitol 1 g/kg. After treatment began, an ophthalmological examination showed unequal pupil diameters, 5 mm right and 3 mm left, with bilateral papilledema with blurred disc margins. Repeated brain CT angiography showed multiple hypodensities associated with diffuse brain edema with a midline shift of 5 mm and impending right uncal herniation. Due to clinical and radiological findings of brain edema, first-tier elevated ICP therapy was initiated, comprising medical neuroprotection with osmotic, sedative, and inotropic therapies: head elevation at 30°, adequate sedation, target arterial partial pressure of CO_2_ (PaCO_2_) of 35–40 mmHg, target serum sodium of 145–155 mmol/L with 3% NaCl infusion, targeted mean arterial blood pressure (MAP) above 60 mmHg, and normothermia. Vasculitis treatment was also started, and she received her first intravenous immunoglobulin (IVIg) of 1 g/kg and was started on induction intravenous methylprednisolone at 30 mg/kg/day for 5 days. The patient then continued maintenance 2 mg/kg/day methylprednisolone in addition to intravenous 250 mg/m^2^ cyclophosphamide monthly for 6 months.

**Fig. 1:**
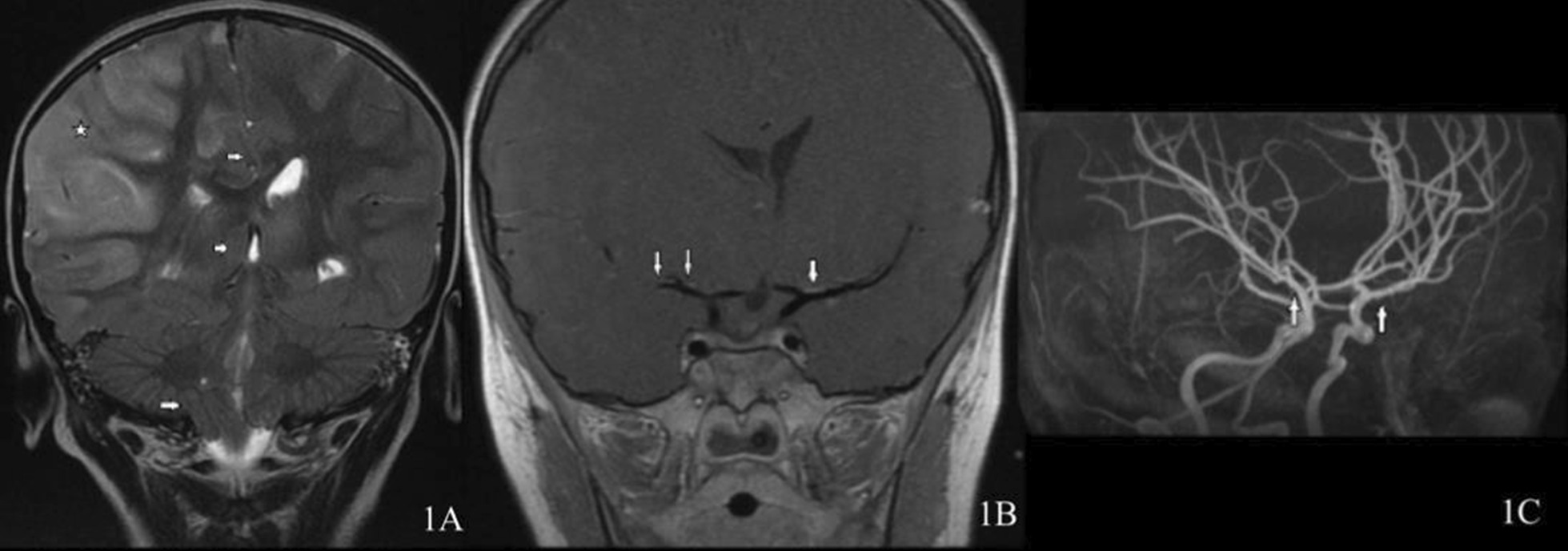
**A** Coronal T2 showing diffuse cortical swelling with T2 high signal intensity involving a large portion of the right cerebrum (star) along with a subsequent midline shift to the left, right uncal, right subfalcine, and tonsillar herniation (arrows). **B** Vessel wall image showing mild vessel wall enhancement involving the middle cerebral arteries (arrows). **C** Magnetic Resonance Angiography (MRA) showing minimal irregularity involving the M1 and M2 segments of both the middle cerebral arteries (arrows)

On HD 4, magnetic resonance angiography/venography (MRI/MRA/MRV) (Fig. 1A–C) showed significant changes in herniation. With acute status epilepticus presentation and bilateral vessel involvement at onset, she was diagnosed with APP-cPACNS. Despite first-tier neuroprotective measures, she was still showing signs of refractory elevated ICP with clinical and radiological signs of herniation. As such, second-tier therapy was commenced, and she underwent an emergency right frontal decompressive hemicraniectomy, with intraparenchymal ICP monitor insertion. A simultaneous brain biopsy and right upper abdomen quadrant implantation of a bone flap was also performed. Intraoperatively, the ICP ranged from 30 to 35 mmHg. The patient was shifted back to the PICU, where medical therapy and treatment of elevated ICP were continued, with a target ICP < 20 mmHg, which was successfully achieved 17 hours later by a thiopental-induced coma. Histopathology showed neutrophil vacuolation. There were few mitotic figures in the white matter and gliosis with prominent clasmatodendrosis.

**Fig. 2:**
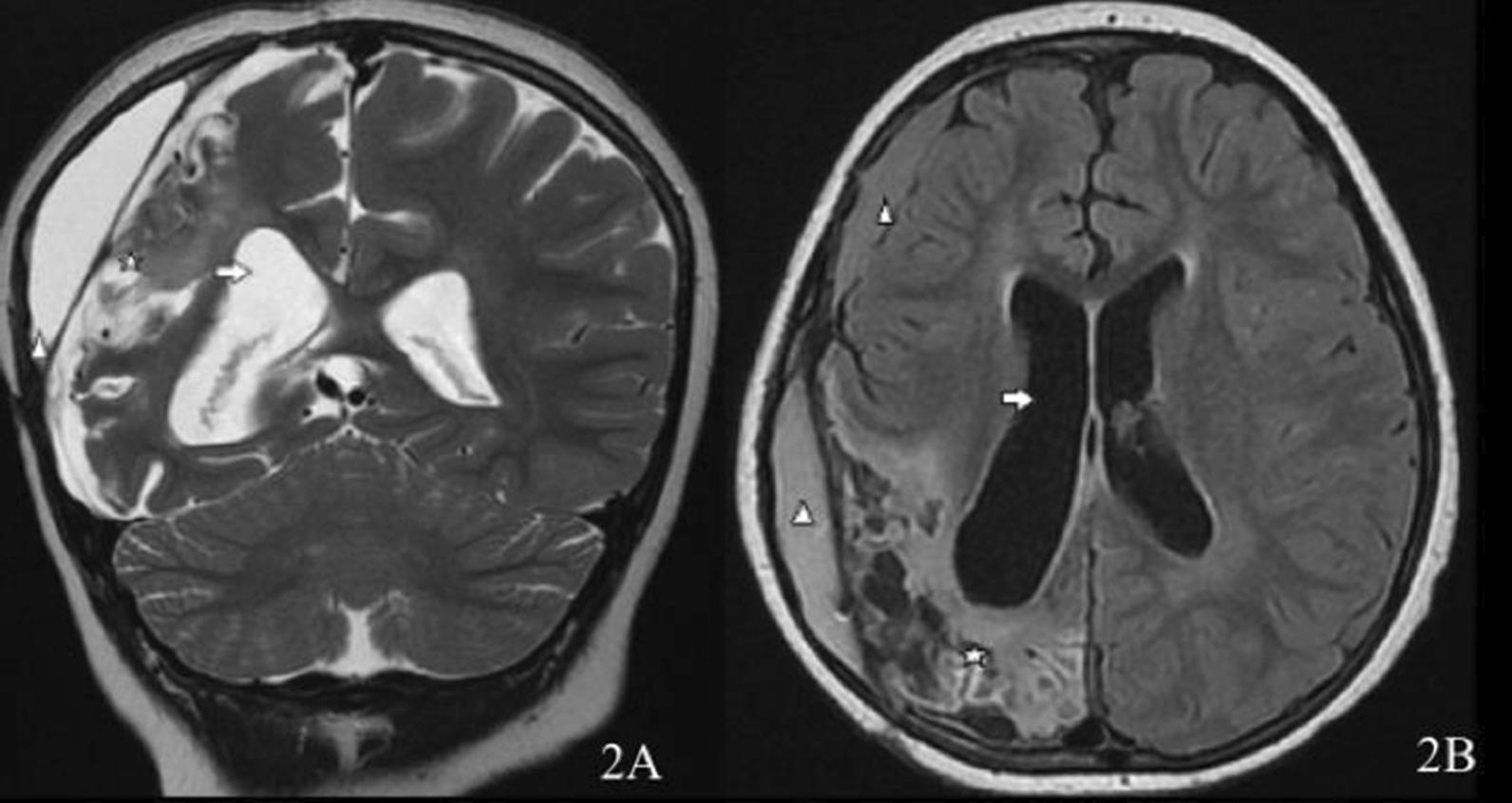
**A** Follow-up coronal T2 revealed right cerebral multifocal (mainly parietal and occipital) encephalomalacia (star) with *ex vacuo* dilation of the ipsilateral lateral ventricle (arrow). There were also small anterior and slightly larger posterior right pericerebral extra-axial collections (arrowhead). **B** Follow-up axial FLAIR revealed right posterior cerebral encephalomalacia (star) with *ex vacuo* dilation of the ipsilateral lateral ventricle (arrow)

On HD 6, the immune therapy was intensified. She received plasmapheresis daily for a total of five sessions, in addition to the maintenance intravenous methylprednisolone. On HD 15, the sedation and thiopental-induced coma were gradually tapered off, and the patient was extubated after clinical and radiological improvement. She was transferred out of the PICU to a high-dependency unit. On HD 60, she underwent cranioplasty using the autologous bone flap from the abdomen. On HD 81, she was discharged conscious and alert, with fluent speech and intact comprehension, and sat independently, but could not walk. She also had oropharyngeal agnosia evident by moderate-to-severe weakness in lingual movement, weak oral bolus manipulation, and delayed oral swallow initiation with oral residue, and laryngeal excursion to palpation was inconsistent and delayed with reduced laryngeal range of motion. She was discharged on levetiracetam, phenytoin, aspirin, cyclophosphamide, and prednisolone. Follow-up MRI/MRA/MRV 6 months later showed marked improvement of vascular enhancement (Fig. 2A and B). Serial monthly clinical follow-ups in an outpatient clinic by multiple teams showed gradual progressive improvement allowing her to regain cognitive and speech abilities. One year after her initial presentation, she had appropriate cognition and speech for her age, normal cranial nerves and cerebellar examinations, and a mild motor deficit (4/5) with left hemiparesis.

## Discussion

To our knowledge, this case is the first report of pediatric APP-cPACNS with substantial CNS inflammation resulting in significantly elevated ICP. The elevated ICP also led to right uncal, right subfalcine, and tonsillar herniation that required decompressive hemicraniectomy.

Decompressive craniectomy (DC) is a treatment for other diseases in children, such as infectious encephalitis, subarachnoid hemorrhage, hemorrhagic and ischemic strokes, and cerebral sinus thrombosis [10–13]. Omay *et al*. [14] and Shah *et al*. [15] both reported a case series of children who underwent decompressive hemicraniectomy due to malignant cerebral infarction, and ischemic stroke, respectively, who had satisfactory outcomes. The benefit of DC over other therapies is the rapid decline in ICP, maintenance of neurologic status, and the ease of performing neurological examination [16].

There is no consensus regarding the ideal timing of DC; however, it may be predicated by neurologic examination, an incidence of neurologic deterioration, the degree of initial ICP, or the refractoriness of ICP to medical treatment [6]. Taylor *et al*. compared medical therapy versus medical therapy combined with DC in children with TBI and found encouraging results, indicating that earlier DC may be advantageous [17]. During the early stages of our patient’s course and despite maximum neuroprotective measures with first-tier therapy and immunological therapy, including induction pulse corticosteroids and IVIg for the inflammation, her neurological status continued to deteriorate and cerebral edema with a midline shift progressed to herniation. The decision to proceed with hemicraniectomy was multidisciplinary to avoid significant morbidity and mortality. Her overall hemodynamic and neurological status improved thereafter.

Due to our patient’s past history of sterile osteomyelitis, an autoinflammatory disorder, predisposing her to other autoinflammatory disorders [18], presenting clinical condition with SE and elevated inflammatory markers, including erythrocyte sedimentation rate (ESR), C-reactive protein (CRP), vWF, and radiographic signs of vasculitis, while excluding other differential diagnoses, inflammatory brain diseases were investigated. She was diagnosed according to the Calabrese criteria [19], which had been adopted for use in children > 1 month and < 18 years of age by Benseler *et al*. [2]. She had a newly acquired diffused neurologic deficit with angiographic evidence of large-vessel cerebral vasculitis. There was no evidence of a systemic underlying condition known to cause CNS vasculitis, as indicated by negative serology and major vessel, renal, and liver venous and arterial ultrasound Doppler.

Our patient presented with SE but exhibited effective neurological, serological, and radiological responses. Her course correlated with a previous report by Celluci *et a*l. [20] stating that patients with AN-cPACNS experience a smaller decline in disease activity following treatment compared with those with AP disease. Seizures at diagnosis also predicted higher levels of disease activity over time.

From the start of the case, the approach of a multidisciplinary team was undertaken. Due to the patient’s radiological finding of two major vascular territory involvement and unique bilateral signal intensity MRI changes, she was treated as having APP-cPACNS, which warranted aggressive antiinflammatory management. No management guidelines were available for cPACNS, but a review by Beelen *et al*. [21] suggested a treatment strategy depending on phenotype, consisting of induction pulse corticosteroid therapy followed by maintenance for 6 months, along with early initiation of monthly cyclophosphamide and IVIg, which we believe provided a favorable overall outcome.

The diagnostic yield of brain biopsies in children presenting with neurological symptoms is around 48.5% [22], but biopsies are crucial to exclude mimics such as malignancy [23]. Elbers *et al*. [24] suggested that nonlesional biopsies can be successful in establishing small-vessel cPACNS. Our patient’s lesional nonviable brain biopsy showed white-matter gliosis and widespread macrophages, with activated microglia but no nodules, and no hemophagocytic histiocytosis (HLH) features. We hypothesize that the diagnostic yield of our patient’s biopsy was lower than expected owing to the nonviability of the sample.

## Conclusions

Decompressive craniectomy might be considered in cases of angiography-positive progressive childhood primary angiitis of the CNS (cPACNS) with elevated ICP refractory to medication. A multidisciplinary approach for the decision of decompressive craniectomy is advised to ensure patient safety and avoid possible morbidities and mortality.

## Supplementary Information

**Additional file 1. **List of laboratory results and medications.

## Data Availability

Please contact the corresponding author for data requests.

## Abbreviations

ICP: Intracranial pressure
cPACNS: Childhood primary angiitis of the central nervous system
CNS: Central nervous system
APP: Angiography-positive progressive
AN: Angiography-negative
DC: Decompressive craniectomy
TBI: Traumatic brain injury
TNF: Tumor necrosis factor
MRI: Magnetic resonance imaging
NSAIDs: Nonsteroidal antiinflammatory drugs
PED: Pediatric emergency department
SE: Status epilepticus
CSF: Cerebrospinal fluid
vWF: von Willebrand factor
CT: Computed tomography
EEG: Electroencephalogram
PICU: Pediatric intensive care unit
HD: Hospital day
PaCO2: Arterial partial pressure of CO_2_
MAP: Mean arterial pressure
IVIg: Intravenous immunoglobulin
MRA/MRV: Magnetic resonance angiography/venography.
RBC: Red blood cell
WBC: White blood cell
ESR: Erythrocyte sedimentation rate
CRP: C-reactive protein
HLH: Hemophagocytic histiocytosis
