# Supplementary material for: Elevated intracranial pressure requiring decompressive craniectomy in a child with progressive primary angiitis of the central nervous system: a case report
Source: J Med Case Rep. 2021 Aug 6;15:418. doi: 10.1186/s13256-021-03005-y (PMC8344202; doi:10.1186/s13256-021-03005-y)
Supplement: Supplementary file 1 — Additional file 1. List of laboratory results and medications. [file 13256_2021_3005_MOESM1_ESM.docx]

Results of laboratory findings:

General:

- WBC highest 29.2 on hospital day 4 (83% neutrophils), hemoglobin and platelet counts were maintained normal throughout her stay.
- Liver function, both synthetic and functional was maintained normal throughout her stay.
- Renal function highest Creatinine was 53 umol/L then normalized.

Infectious:

- Set of blood cultures were collected from central and peripheral samples in sterile manner, cultivated in aerobic medium. Placed in incubator, then sub-cultured in an agar plate for identification and sensitivity.
- Fungal culture from blood and CSF were collected in sterile manner, inoculated in brain heart infusion agar, incubated for 4 weeks.
- All cultures, including blood, urine, CSF, and fungal cultures were non-revealing.
- Brucella titers, brucella PCR, and brucella DNA were negative.
- TB-QuantiFERON from blood, AFB culture from blood and CSF, and TB-PCR from CSF were also done which was negative.
- CMV and EBC PCR from blood were negative.

Immunological:

- Immunological workup included immune globulin levels, oxidative burst assay, lymphocyte markers, primary immune deficiency gene panel which were all negative.

Genetic:

- Genetic work up included whole exome sequencing and whole genome sequencing which were both negative.

Hematological:

- Hemoglobin electrophoresis was negative

Routes of administration, duration of treatment, and doses of all medications that were given during hospital stay and follow-up:

1st admission:

- Intravenous ceftriaxone 50 mg/kg Q12H for 2 weeks
- Intravenous vancomycin 15 mg/kg Q6H for 2 weeks
- Oral sulfamethoxazole-trimethoprim 127.5 mg three times per week
- Oral Aspirin 81 mg daily

2nd admission:

- Intravenous Levetiracetam 40 mg/kg then daily 20 mg/kg Q12H
- Intravenous Phenytoin 20 mg/kg loading then daily 45 mg Q12H
- Intravenous vancomycin 15 mg/kg Q6H for 2 weeks
- Intravenous meropenem 35 mg/kg Q8H for 5 weeks
- Intravenous immunoglobin 10% 1 gm/kg every 2 weeks for 6 weeks then monthly for 1 year
- Intravenous Methylprednisolone 30 mg/kg for 5 days
- Intravenous methylprednisolone 2mg/kg/day divided Q8H for 5 weeks
- Intravenous cyclophosphamide 250 mg/m2 monthly for 6 months
- Plasmapheresis daily for 5 sessions
- Intravenous continuous infusion sodium chloride 3% at 1 ml/kg/hr for 7 days
- Intravenous Epinephrine infusion, highest dose 0.08 mcg/kg/min for 12 days
- Intravenous Midazolam infusion, highest 4 mcg/kg/min for 16 days
- Intravenous Fentanyl infusion, highest 4 mcg/kg/min for 16 days
- Intravenous Thiopental infusion, highest 5 mg/kg/hr for 7 days
- Intravenous Morphine 1 mg Q6H for 14 days
- Intravenous Diazepam 1.8 mg Q12H for 27 days
- Oral Aspirin 81 mg daily
- Oral Gabapentin 275 mg Q12H for 5 months
